# Investigating pianists' individuality in the performance of five timbral nuances through patterns of articulation, touch, dynamics, and pedaling

**DOI:** 10.3389/fpsyg.2014.00157

**Published:** 2014-03-04

**Authors:** Michel Bernays, Caroline Traube

**Affiliations:** ^1^IDMIL/SPCL, Schulich School of Music, McGill UniversityMontreal, QC, Canada; ^2^LRGM, OICRM, Faculté de musique, Université de MontréalMontreal, QC, Canada

**Keywords:** piano, performance, timbre, individuality, expression, articulation, touch, Bösendorfer CEUS

## Abstract

Timbre is an essential expressive feature in piano performance. Concert pianists use a vast palette of timbral nuances to color their performances at the microstructural level. Although timbre is generally envisioned in the pianistic community as an abstract concept carried through an imaged vocabulary, performers may share some common strategies of timbral expression in piano performance. Yet there may remain further leeway for idiosyncratic processes in the production of piano timbre nuances. In this study, we examined the patterns of timbral expression in performances by four expert pianists. Each pianist performed four short pieces, each with five different timbral intentions (bright, dark, dry, round, and velvety). The performances were recorded with the high-accuracy Bösendorfer CEUS system. Fine-grained performance features of dynamics, touch, articulation and pedaling were extracted. Reduced PCA performance spaces and descriptive performance portraits confirmed that pianists exhibited unique, specific profiles for different timbral intentions, derived from underlying traits of general individuality, while sharing some broad commonalities of dynamics and articulation for each timbral intention. These results confirm that pianists' abstract notions of timbre correspond to reliable patterns of performance technique. Furthermore, these effects suggest that pianists can express individual styles while complying with specific timbral intentions.

## 1. Introduction

Musical performance holds a crucial role in the art and experience of music. Classical performers in particular can shine their own light upon the composed work and express their creativity. Accordingly, an extensive, empirical body of knowledge has been developed amongst musicians with respect to the art and technique of performance. Notably, it has been so for the piano in the few centuries since the instruments' inception. Guidelines about technique, gesture, touch, and mental approach were provided by teachers and pedagogues in the aim of helping pianists develop their own “sound” and musical expression (Hofmann, 1920; Neuhaus, 1973; Fink, 1992). Individual pianist development is then shaped and oriented by the teacher and the piano school (e.g., Russian, German or French) he/she abides by Lourenço (2010).

A large body of research has been devoted to exploring expressive piano performance, by examining the general performance parameters (in opposition to the musical parameters of pitch, harmony or rhythm Rink et al., 2011) that pianists can use as expressive devices. Expressive control parameters of timing and amplitude have been the most explored by far, for their salience among the performance parameters that pianists can vary and their effect on the perception of emotional expression (Bhatara et al., 2011), and for the relative accessibility of their measurement (e.g., with MIDI digital recording pianos or with acoustical analysis Goebl et al., 2008). They were revealed to follow broad, common expressive strategies that depend on other musical factors. In particular, expressive deviations from the score in timing and dynamics were shown to follow common patterns related to the musical structure (phrasing and local accents) (Repp, 1992; Shaffer, 1995; Parncutt, 2003). Moreover, overlap durations in *legato* articulation were shown to depend on register, tempo, interval size and consonance, and position in an arpeggio (Repp, 1996b), while the melody lead effect (i.e., the melody note in a chord played slightly earlier) was shown as an artifact of the dynamic accentuation of the melodic voice, thus correlated to amplitude (Goebl, 2001). Different computational models of expressive performance were developed (Widmer and Goebl, 2004), based on structurally guided rules of expressive timing and dynamics (MIDI parameters). These rules could be defined in different ways (De Poli, 2004): explicitly (as heuristics), through analysis-by-measurement (Todd, 1992 e.g.,) or analysis-by-synthesis (Friberg et al., 2006); or implicitly, by machine learning, from an input learning set of recorded human performances (Widmer et al., 2009).

However, within the constraints imposed by these structural rules of timing and dynamics, there remains enough space for pianists to bring out their individual expression in performing a piece, as advocated in piano pedagogy and performance. Indeed, idiosyncratic patterns, that would differ between pianists yet remain consistent for each one, were identified below general rules, in the following expressive techniques: in chord asynchronies and melody lead (Palmer, 1996); in temporal deviations around the frame defined by the musical structure (so long as these deviations remain within an “acceptable” range) (Repp, 1990, 1992, 1998); in articulation, for which the general, common trends in overlap durations were obscured by considerable inter-individual variations (Bresin and Battel, 2000); and also in sustain pedal timing (Heinlein, 1929; Repp, 1996c, 1997). On the other hand, in Repp's (1996a) study of 30 performances by 10 graduate students of Schumann's “Träumerei,” the expressive dynamics (MIDI velocities) did not appear as a clear bearer of individual differences, yet tended to be more consistent in the repeated performances by each pianist than between performers.

Expressive features of both timing and loudness were used successfully for automatic identification, with machine learning models, of the performer in MIDI (Stamatatos and Widmer, 2005) or audio (Saunders et al., 2008; Wang, 2013) recordings of piano performances. Meanwhile, temporal deviations were identified as individual fingerprints in non-expressive scale playing, yet could not be perceived by human listeners (Van Vugt et al., 2013), indicating that a fine-grained level of individuality in piano performance resides below the level of expressive timing.

More generally, in a review paper, Sloboda (2000) examined the performers' abilities to generate expressively different performances of the same piece of music according to the nature of intended structural and emotional communication, and described how some of these abilities have been shown to have lawful relationships to objective musical and extra-musical parameters. With respect to other keyboard instruments, it was also shown that local tempo variations, onset asynchronies, and especially articulation (overlaps) were highly individual parameters of expressive performance in Baroque organ music (Gingras et al., 2011). How these findings relate to piano performance, however, is a non-trivial issue.

Individuality in expressive piano performance was also examined in light of musical gestures, i.e., timing and dynamic patterns, whose occurrences, distribution and diversity could characterize the individual expressive strategies in 29 case-study performances of Chopin's Mazurka, op.24 no.2 (Rink et al., 2011). Conversely, literal pianists' gestures (body motion, finger movements) in expressive piano performance were shown as highly idiosyncratic, although related to the musical and rhythmic structure of the piece (MacRitchie, 2011; MacRitchie et al., 2013). Likewise, finger kinematics were shown as idiosyncratic enough for performers to be accurately identified, with neural network classifiers, from their finger movements and accelerations during attacks and key presses (Dalla Bella and Palmer, 2011).

However, such pianistic gestures hold other functions (ancillary, figurative) than the actual, effective sound production (Cadoz and Wanderley, 2000). Consequently, their idiosyncratic nature does not necessarily translate into an idiosyncratic expressive sound production—the main concern of this article. Thus, this study only considers the effective gestures applied by pianists to the keyboard and pedals.

Among the expressive musical attributes available to pianists other than sheer timing and loudness, timbre holds a crucial expressive role (Holmes, 2012), which has been widely acknowledged within the pianistic community (Bernays, 2012). Usually envisioned as the inherent characteristic of a sound source or instrument, timbre is also considered by pianists as the subtle quality of sound that they can control through the expressive nuances of their performances. However, it has long been debated whether pianists can actually control timbre as a sound quality of performance. Scientific studies concluded long ago that controlling piano timbre on a single key was limited by the mechanical constraints of the action to sheer keystroke velocity, and thus inseparable from intensity (Hart et al., 1934). The influence of contact noises however (especially finger-key contact) on the timbre of a single piano tone was demonstrated (Goebl and Fujinaga, 2008), suggesting that the type of touch can bear an influence on the timbre of a single tone (Goebl et al., 2005). Yet even so, keyboard control over the timbre of a single tone remains quite limited.

But in a polyphonic, musical context, the expressive performance features of articulation, touch and pedaling can govern subtle tone combinations, in the timing and dynamic balance (*polyphonic touch*) of notes in a chord and in melodic lines (Parncutt and Troup, 2002). Composite timbres thus arise which are, in essence, performer-controlled (Bernays, 2013). Pianists' expressive intentions can thus be conveyed through specific timbral nuances (Sándor, 1995), to the vast palette of which an extensive vocabulary, including numerous adjectival descriptors, has been associated (Bellemare and Traube, 2005; Cheminée, 2006). However, the precise technique and ways of production of piano timbre nuances have generally been subdued to abstraction, mental conception, imitation and aural modeling (Woody, 1999) in piano pedagogy and treatises (Kochevitsky, 1967; Neuhaus, 1973). This abstract approach to teaching the production of piano timbre, in combination with the focus on personal expression, thus suggest that pianists may employ individual, idiosyncratic expressive performance strategies toward producing a specific timbral nuance—whose understanding according to its verbal descriptor may also vary slightly between pianists.

In order to explore pianists' individuality in the production of timbral nuances, piano performance has to be measured and quantified with the high precision required for identifying the subtleties of expressive performance employed in controlling timbre. In the absence or rarity of high-precision measurement tools for piano performance, the intricacies of timbre production have essentially remained out of the reach and/or concern of piano performance studies—with the exception of Ortmann's (1929) investigation, with the help of cumbersome mechanical apparatus, of the relations between piano touch and timbre on a single tone. Going further, with the high-accuracy Bösendorfer CEUS digital piano performance-recording system, this study explores pianists' individuality in the production of piano timbre in a polyphonic, ecologically valid musical context.

Furthermore, the verbalization of piano timbre was studied quantitatively (Bernays and Traube, 2011), according to judgements of semantic similarity between the 14 descriptors of piano timbre most cited by pianists in Bellemare and Traube (2005). These evaluations were mapped into a semantic space, whose first two, most salient dimensions formed a plan in which descriptors were grouped in five distinct clusters—which was confirmed by hierarchical cluster analysis. In each cluster, the descriptor judged the most familiar was selected. The five most familiar, diverse and representative timbre descriptors thus highlighted—**dry, bright, round, velvety**, and **dark**—appear (in that order) along a circular arc in the semantic plan. These five descriptors defined the timbres for which to seek out individual patterns of production between several pianists.

This method of searching for production patterns in performances driven by verbal descriptors has been employed in studies of emotions in music performance. Verbal descriptors of emotion in music were first categorized by Hevner (1936), in eight groups arranged by similarity in a circular pattern. Studies of emotional expression in music performance have used a subset of emotional descriptors—taken either from Hevner's categories or from the verbal descriptors of basic emotions in a general context (Ekman, 1992)—in order to drive the performers' intentions. Emotional descriptors among “happiness,” “anger,” “sadness,” “tenderness,” “fear,” “solemnity,” as well as emotionally “neutral” (for comparison), were for instance used as emotional instructions to several performers playing various instruments in Gabrielsson and Juslin (1996), Juslin (1997), Juslin and Laukka (2003) (a meta-study), and Quinto et al. (2013). Among other goals, correlations were identified between these emotional expressions and performance parameters of dynamics, tempo, timing, articulation, as well as acoustical features of tone and timbre. In more details, both intensity and tempo were positively correlated with arousal (sadness, tenderness vs. anger, happiness), and high variability in intensity was associated with fear. *Legato* articulation was found to reflect the expression of tenderness or sadness, while a *staccato* articulation expressed happiness. Individual differences between performers in encoding each emotion were also mentioned, yet hardly detailed. In particular, Gabrielsson and Juslin (1996) concluded that the performance rules for communicating emotions depend on the instrument, the musical style, and the performer, as well as on the listener. Meanwhile, Canazza et al. (1997) used sensorial-type adjectives (bright, dark, hard, soft, heavy, light, and normal) as instructions for expressive clarinet performances of the same musical piece, and identified sonological characteristics of these emotions.

Regarding the piano, Madison (2000) explored the expression of happy, sad, angry, and fearful emotions in performances by three pianists, and identified correlations between emotional expression and the degree of variability in patterns of timing, articulation and loudness.

Furthermore, Ben Asher (2013) developed and trained a machine learning algorithm to automatically retrieve in real time the musical expression and emotional intentions in piano performance from the gestures of pianists. The training set used verbal descriptors of basic emotions to drive the performances, and the pianists' high-level gestures were automatically identified from kinaesthetic data.

However, we may argue that the verbal descriptors of basic emotions used in such studies are not comparable, in the context of music performance and pedagogy, with the verbal descriptors of piano timbre nuances we are using in this article. Indeed, the vocabulary that is used by musicians and music teachers to verbally define and communicate the emotional qualities of music in performance is based on more complex or indirect metaphors and analogies, most often attempting to connect emotions and music performance through their shared motor-affective elements and motional aspects, for instance with reference to bodily gesture or vocal intonations (Woody, 2002). Those may be more effective in triggering appropriate actions from the performer, whereas the vocabulary of basic emotions may be comparatively better suited to music perception. On the other hand, the vocabulary describing musical timbre was demonstrated as consensual and meaningful (Faure, 2000). In particular, the vocabulary of verbal description of piano timbre forms a specialized lexicon that holds a distinctive meaning within the context of piano performance (Cheminée, 2006), and was shown as familiar to pianists in a musical context, and largely shared among them (Bellemare and Traube, 2005).

The first research question explored in this study is whether individuality in piano performance manifests itself in the gestures applied by pianists on the keyboard, and if so, which descriptive performance features of dynamics, touch, articulation, and pedaling determined from high-resolution key/pedal position and hammer velocity tracking can reveal individual piano performance patterns. Yet the main research question that we wish to investigate is whether individuality in piano performance, if highlighted by specific performance features, arises in different patterns between performances of different timbral nuances, i.e., whether the characteristics of piano performance that would prove idiosyncratic in comparing different pianists vary depending on the timbral nuance performed.

## 2. Materials and methods

In order to explore pianists' individuality in the expressive production of piano timbre nuances in a musically relevant framework that could mirror a genuine musical experience, the study was designed with respect to the following steps: selection of the five verbal descriptors **dry, bright, round, velvety** and **dark** as timbral instructions for which to explore performance idiosyncrasies; conception of musical pieces to be expressively performed according to these different timbral nuances; use of non-invasive, high-accuracy piano performance-recording equipment; recording of timbre-colored performances; and extraction therein of meaningful piano performance, articulation, touch and pedaling descriptors.

### 2.1. Musical pieces

**Figure 1:**
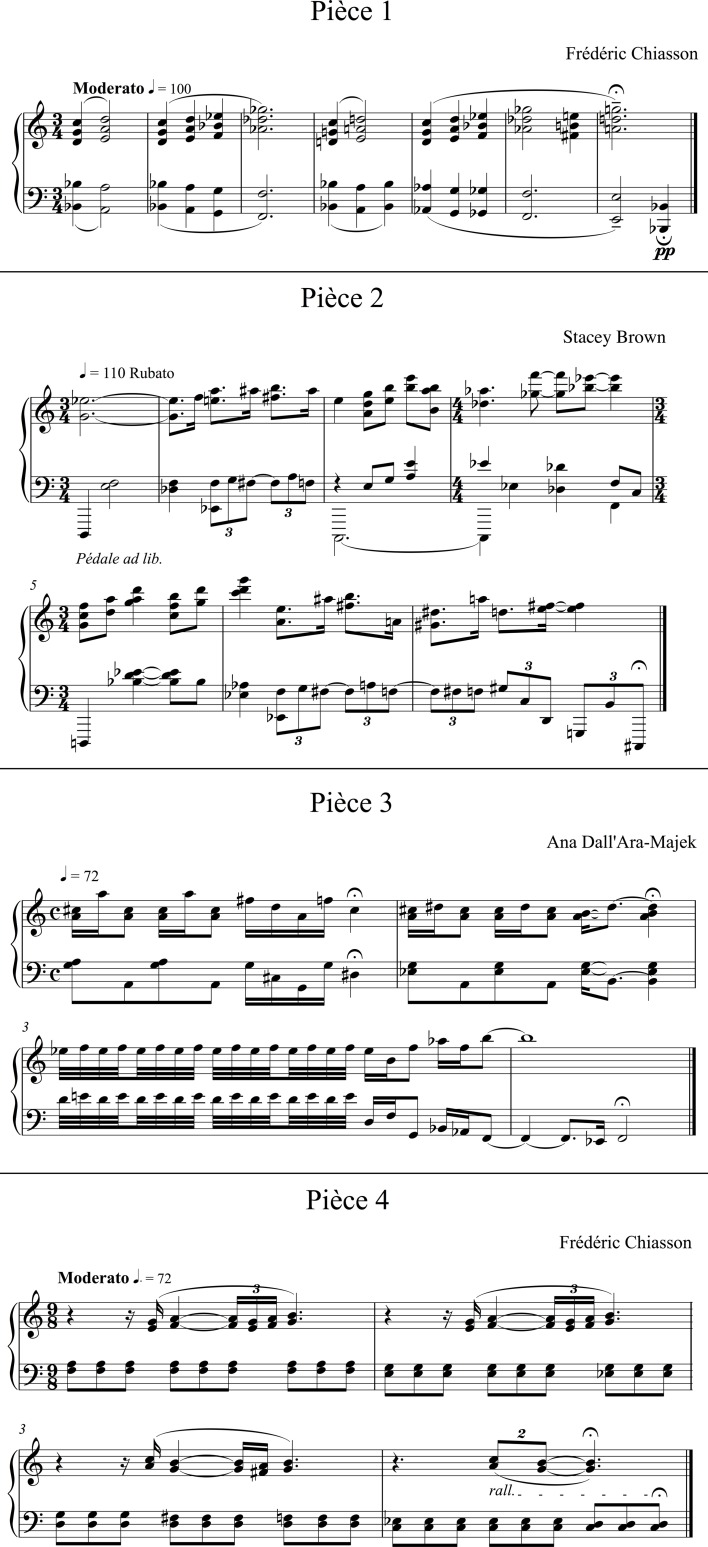
**Scores of the four pieces composed and selected for the study**.

In order to set a musical context adequate to expressive timbre production in performances, four short solo piano pieces were selected, among 15 specially composed for the study following instructions on the timbral nuances to be expressed (cf. Figure 1). Each selected piece could allow for a meaningful, consistent-throughout expression of each of the five timbral nuances, and featured many aspects of piano technique that we wanted to explore. Each just a few bars long (from 4 to 7, with different meters), their duration at score tempo ranged between 12 and 15 s.

### 2.2. Equipment

To investigate the fine-grained nuances of pianists' performance control and touch that let them express different timbral nuances and could reveal idiosyncratic approaches, highly precise data were required from which to thoroughly assess the intricacies of key strokes. In this aim, we had the opportunity to use the Bösendorfer CEUS piano digital recording and reproducing system. Vastly improving upon the MIDI-based SE reproducing piano, the CEUS system is designed to both record with high accuracy the actions of a pianist on the keyboard, and to reproduce the performance faithfully, with solenoids that activate each key and pedal to mirror the original, recorded performance. In this study, the CEUS system was only used for its recording abilities. Equipped with optical sensors behind the keys, hammers and pedals, microprocessors, electronic boards, and a computer system, it can indeed track key and pedal positions and hammer velocities at high resolution (8-bit) and high sampling rate (500 Hz). The system we used was embedded in the Imperial Bösendorfer Model 290 grand piano installed at BRAMS (International Laboratory for Brain, Music and Sound Research, Montreal, Canada) in a dedicated recording studio.

### 2.3. Participants

Four pianists participated in the study. They are further referred as pianists A, B, C and D. All four had extensive professional experience and advanced-level piano performance diploma. Pianist A is a 30-year old French female. She studied piano and played professionally in France and Belgium. Pianist B is an 54-year old Italian male. He studied piano in Switzerland and Italy, and played professionally in several countries. Pianist C is a 46 year-old French-Canadian male. He studied piano and played professionally in the Quebec province, Canada. Pianist D is a 22-year old French male. He studied piano and played professionally in France and Canada.

### 2.4. Procedure

Each participant had received in advance the scores of the pieces and the timbral instructions, and was given time to practice. Rehearsal sessions were allotted on the Bösendorfer piano, to allow for familiarization with the instrument and the room. The timbral instructions were provided with only the five adjectival descriptors. The participants confirmed their familiarity with each descriptor as a piano timbre nuance. They were asked to perform each of the four pieces, with each of the five timbres. Three such runs of 20 performances were conducted successively—twice in an order of pieces and timbres chosen by the participant, and once in randomized forced order—so as to get three performances for each condition (piece × timbre). The participants were allowed to immediately replay a performance if they considered the previous try unsatisfactory. Each of the 60 performances per participant was recorded through the CEUS system. We thus collected 240 CEUS boe-format recordings of 4 pianists performing 4 pieces with 5 different timbres, 3 times each.

### 2.5. Performance analysis and extraction of performance features

In order to extract meaningful piano performance and touch features from CEUS-acquired data, the PianoTouch Matlab toolbox was specifically developed (Bernays and Traube, 2012). From the high-frequency, high-resolution key/pedal positions and hammer velocities, note and chord structures were retrieved, and an exhaustive set of quantified features spanning several broad areas of piano performance and touch were computed:

- Dynamics and attack: maximum hammer velocity (MHV), maximum key depression depth (Amax) and their relations (ratio of their values, respective timing); attack durations (related to instants of both MHV and Amax), speeds (ratio of MHV or Amax to duration) and percussiveness (pressed vs. struck touch) (Goebl et al., 2005; McPherson and Kim, 2011)
- Articulation: sustain and release durations, synchrony of notes within chords (melody lead; duration, rate and amount of asynchrony at onset and offset), intervals and overlaps between chords (inter-onset and offset-to-onset, overlap durations, number and corresponding amount of depression, with respect to all chords, same-hand chords and other-hand chords)
- Detailed use of the soft and sustain pedals during each chord: duration of use, of full depression and of part-pedaling, timing with regard to chord onsets and offsets, depression (average, maximum, depth at chord onset, offset and MHV).

Each note was thus described by 46 features. Averages and standard deviations of these note features per chord were calculated as chord features. With the addition of 76 chord-specific features, each chord was described by 168 features.

For each performance, averages and standard deviations of chord features were calculated over all the chords in the performance. Only the chord features whose averaging could provide a meaningful description of a performance were conserved. For instance, the absolute instants of chord onsets, while useful in describing a chord, are meaningless in and of itself when averaged over a performance. However, such chord features were indispensable as building blocks for calculating other chord features (e.g., synchrony or attack speed) that can be meaningfully averaged as performance features and compared between different performances. Performance features were thus given by the averages and standard deviations per performance calculated for 100 relevant chord features (out of 168). With the addition of the number of chords and total number of notes per performance, each performance was thus described by 202 performance features.

Moreover, performances features were also calculated over only the chords played with the left hand and over only the chords played with the right hand, and differences in average performance feature values between hands were determined. The 32 pedaling features per chord were not considered in this context. Excluding their averages and standard deviations per performance (64 performance features), there remained 138 performance features to describe each performance with regard to left-hand chords only, to right-hand chords only, and to the differences between hands.

In total, over the four different chord groupings per performance (all chords, left-hand chords only, right-hand chords only, and differences between hands), 202 + 138 × 3 = 616 performance features were calculated to characterize each of the 240 recorded performances.

## 3. Results

### 3.1. Pianists' overall individuality

First, in order to obtain an overall picture of each pianist's individual and idiosyncratic patterns of articulation, touch, dynamics and pedaling in all the performances whose key and pedal depressions and hammer velocities were recorded, the performance features that could prove characteristic of one pianist's performances in contrast with the others' were sought out over the whole dataset of 240 performances of four different pieces by four pianists highlighting five different timbral nuances.

#### 3.1.1. Statistical method

Statistical analysis of variance was conducted, with regard to the 616 performance features describing each performance, over the 240-performance dataset. Three-Way repeated-measures ANOVAs were performed for each performance feature (as dependent variable), with the performer as random factor, and timbre, piece, and repetition (of the same experimental condition) as fixed-effect factors. Two factors were considered for assessing pianists' overall individuality in performance: the random factor of performer, and the fixed-effect repetitions. Indeed, for a performance feature to reveal pianists' individuality, the effect of the performer has to be significant (i.e., rejecting with at least 95% confidence the null hypothesis of equal variance between pianists), but the feature must also remain consistent between repetitions (i.e., no significant differences between repetitions at the 5% level).

For these two factors, the assumptions required by the ANOVA were tested. For the between-subject factor, assumptions of normal distributions (Kolgomorov–Smirnov test) and homoscedasticity (Levene's test) were tested. In the cases where the ANOVA was significant at the 5% level in rejecting the null hypothesis of equal variance between performers but the assumptions were not met, the non-parametric, one-way Kruskal–Wallis rank analysis of variance was also run, to control for possible type I errors (i.e., to confirm or invalidate significance, depending of the significance at the 5% level of the Kruskal–Wallis test). Effect sizes and statistical power were also calculated. For the repetition, within-subject factor, the Huynh–Feldt correction was applied to the degrees of freedom in order to compensate for possible violations of sphericity (as assessed with Mauchly's test).

With this method, 159 performance features were revealed as both consistent (i.e., not significantly different at the 5% level) between repetitions, and different between the four pianists (i.e., significant at the 5% level in rejecting the null hypothesis of equal variance between them).

#### 3.1.2. Reduced performance space

Principal Component Analysis was applied over the dataset of 240 performances to those 159 selected performance features. Indeed, those are the performance features that were shown to highlight consistent, significant difference between the four performers (within the limits of statistical accuracy, as will be discussed in Section 4); the excluded performance features would only impede the visualization of differences between performers. The values of the selected performance features were normalized (Z-scores per feature over the 240 performances), in order to assess their relative values, so as to enable comparisons between features on an equivalent scale, and so that each pianist's individuality according to each feature could be expressed as a relative deviation from the overall average (Wöllner, 2013). The PCA procedure aimed at defining reduced performance spaces whose dimensions would correspond to the first few principal components and that would illustrate the aspects and features of performances most relevant in highlighting the performers' individuality. The number of principal components required to explain a sufficient part of the variance in the input dataset determined the number of dimensions of each reduced performance space, and the meaning of each dimension in terms of the performance features it represents most saliently was explored in its loadings (i.e., the weights associated to each feature in the linear combination which forms the principal component).

**Figure 2:**
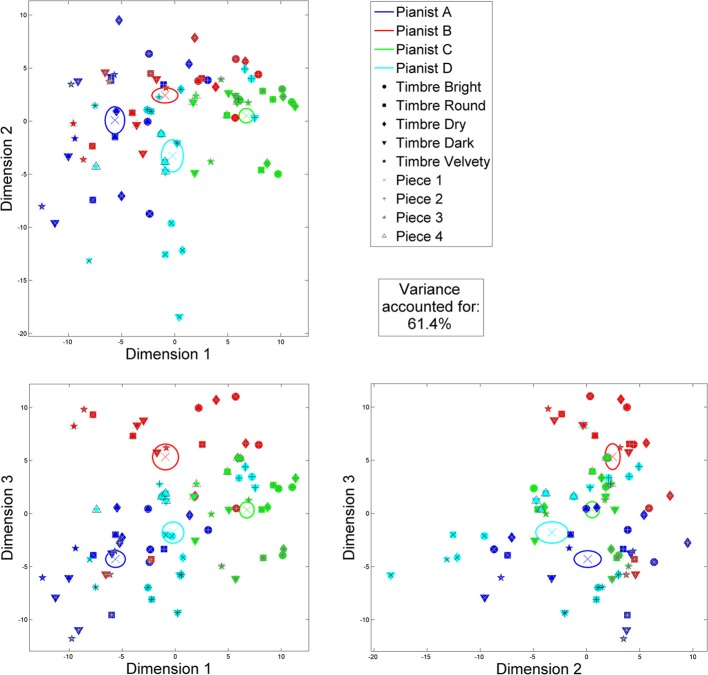
**Reduced 3-dimension performance space by PCA and varimax factor rotation applied to 159 significant performance features over the 240-performance dataset: planar projections**. For a clearer representation, only the averages of the three repetitions of same-condition performances are plotted. Averages per pianist are indicated by colored crosses, and ±1 SE with ellipses.

The first three principal components of the PCA applied to the 159 selected performance features over the 240-performance dataset account for 61.4% (31.97, 15.56, and 13.87% respectively) of the input variance. Varimax factor rotation (Kaiser, 1958) was then applied to these first three PCA loadings, in order to optimize the weightings of performance features between the loadings. The average positions (over the three repetitions) of same-condition (pianist, piece and timbre) performances, according to their coordinates in these three rotated dimensions, are presented in Figure 2.

According to the varimax-rotated loadings, the first dimension corresponds to performance features of attack and dynamics: hammer velocity and its variations between chords, attack speed, duration, and percussiveness, and key depression depth. For the second dimension, the loadings are principally attributed to performance features of chord, note, sustain, and overlap durations, as well as right-hand note offset timing in chords, and inter-onset intervals. Finally, the third dimension essentially accounts for performance features of articulation: number of overlapping chords, *legato* vs. *staccato* articulation, interval between chords, and melody lead.

In the performance space thus defined, some idiosyncratic, consistent performance tendencies of the four pianists are revealed. Pianist A shows the lowest dynamics and longest/slowest attacks, and the most *legato* articulation. On the other hand, Pianist C uses the highest dynamics and fastest/shortest attacks, while employing rather short notes and detached articulation. As for Pianist B, he tends to employ short notes with the most *staccato* articulation. Finally, Pianist D tends to let keys depressed the longest, with a *legato* articulation.

#### 3.1.3. Descriptive performance portrait

Now, in order to obtain a more precise account of the four performers' idiosyncrasies as reflected by specific performance features, the most salient, relevant, meaningful and non-redundant features among those significant between performers (and consistent between performers' repetitions) were sought out. For this aim, the significant performance features were first divided into the four broad, technically independent categories of: (1) dynamics and attack, (2) articulation, (3) soft pedal, and (4) sustain pedal. Correlations were calculated between all the significant performance features pertaining to the same category, and hierarchical clustering was used to regroup the most correlated (thus redundant) features into a same cluster. For each cluster identified in each category, the most significant, meaningful and interpretable performance features could then be selected, while minimizing the loss of information from the other discarded features. This process allowed for selecting a minimal number of performance features to highlight the differences between the four pianists and draw a unique portrait of each pianist's performing individuality.

**Figure 3:**
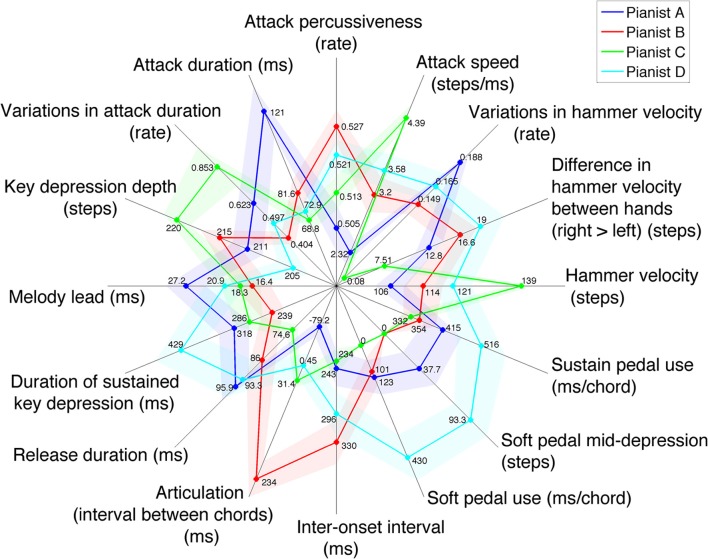
**Kiviat chart of the 16 performance features giving a minimal and unique description of four pianists' individual performance patterns**. Z-scores per pianist are plotted for each feature with colored dots, with the corresponding unnormalied values indicated alongside. The four colored, dot-linking closed lines portray each pianist's performing style. Shades around each closed line show the ±1.96 SE. intervals (95% confidence interval).

Following this method, 16 performance features were selected among the 159 previously identified as significantly differing between performers and consistent between repetitions. With these 16 selected performance features, a minimal, unique and meaningful description of each pianist's individuality in the 240 recorded performances is obtained. The overall performance portraits of each pianist according to these 16 performance features are presented in Figure 3.

The 16 selected performance features are described below, and the corresponding statistical scores (ANOVA F-ratio, *p*-value and effect size) are provided.

- Hammer velocity [*F*(3, 19.66) = 13.141, *p* < 10^−4^, η^2^ = 0.354]: maximum hammer velocity for each note, as directly measured by the piano sensors; as a direct correlate to intensity, it makes for a descriptor of dynamic level. Values are indicated in 8-bit steps (from 0 to 250).
- Difference in hammer velocity between hands [*F*(3, 15.79) = 3.611, *p* = 0.037, η^2^ = 0.234]: compares hammer velocity between notes played with the right hand and notes played with the left hand, and can thus underline a dynamic emphasis with one hand.
- Variations in hammer velocity [*F*(3, 17.01) = 16.603, *p* < 10^−4^, η^2^ = 0.570]: describes the range of hammer velocities reached in each performance; values are indicated as ratios of deviation from the average hammer velocity.
- Attack speed [*F*(3, 19.48) = 15.413, *p* < 10^−4^, η^2^ = 0.420]: mean attack speed (in 8-bit steps per ms) from the beginning of key depression to the maximum depression reached in the note; although this feature is highly correlated with hammer velocity, some differences can occur, as hammer velocity is defined by the instant key speed at hammer launch instead of the mean attack speed over the keystroke.
- Attack percussiveness [*F*(3, 2.38) = 15.496, *p* = 0.043, η^2^ = 0.226]: an evaluation of keystroke acceleration during attack, this feature indicates the convexity of the keystroke curve: the higher the early key acceleration, the more concave the keystroke curve, and the more percussive the attack—as it corresponds to a key struck rather than pressed (Goebl et al., 2005); a value of 0.5 would indicate a linear attack (on average), while values over 0.5 suggest a concave, more percussive touch.
- Attack duration [*F*(3, 14.61) = 6.836, *p* = 0.004, η^2^ = 0.333]: time interval (in ms) from the start of keystroke to its maximum depression; although highly negatively correlated to attack speed (the faster the attack, the shorter its duration), it also depends on nuances of articulation and touch at note onsets, including attack percussiveness and key depression depth.
- Variations in attack duration [*F*(3, 11.79) = 4.405, *p* = 0.027, η^2^ = 0.217]: variations of attack durations between chords and in time; values are indicated as ratios of deviation from the average attack duration.
- Key depression depth [*F*(3, 17.69) = 7.945, *p* = 0.001, η^2^ = 0.320]: indicates how deep (close to the keybed) each key gets depressed for each note; values are given in 8-bit steps.
- Melody lead [*F*(3, 6.38) = 32.003, *p* = 3.1 · 10^−4^, η^2^ = 0.095]: time interval (in ms) describing the advance of the first note of a chord on the others; as despite its description in terms of timing, melody lead is essentially a velocity artifact of the dynamic accentuation of the melody note (Goebl, 2001), it is thus a feature of polyphonic, dynamically differentiated touch. Despite a small effect size, the significance of this feature is supported a high statistical power (π = 0.969), and it was found to be independent from other features of attack, touch and articulation according to hierarchical clustering analysis.
- Duration of sustained key depression [*F*(3, 11.99) = 4.744, *p* = 0.021, η^2^ = 0.201]: time (in ms) for which a key is held depressed, after attack and before release; although tempo can bear an effect on this feature, it is also a descriptor of articulation strategies.
- Release duration [*F*(3, 18.41) = 3.545, *p* = 0.035, η^2^ = 0.148]: time (in ms) taken for releasing the key (from the start of the key moving up to its reaching rest position); this feature essentially accounts for articulation: a note released slowly (thus slowed by the finger) may probably overlap with the next.
- Articulation (interval between same-hand chords) [*F*(3, 8.84) = 10.929, *p* = 0.002, η^2^ = 0.565]: time interval (in ms) from the end of a chord or single note to the start of the next one played with the same hand; negative values indicate *legato*, positive values *staccato*.
- Inter-onset interval [*F*(3, 13.28) = 6.151, *p* = 0.008, η^2^ = 0.168]: time (in ms) elapsed between the start of two consecutive chords; this feature essentially serves as a descriptor of average tempo (Dixon, 2001).
- Soft pedal use [*F*(3, 14.94) = 6.356, *p* = 0.005, η^2^ = 0.316]: average time (in ms) during which the soft pedal is depressed while a chord is being played.
- Soft pedal mid-depression [*F*(3, 6.79) = 16.249, *p* = 0.002, η^2^ = 0.380]: indicates the amount (in 8-bit steps) of part-pedaling during chords; the higher the value, the more the soft pedal was kept only partially depressed.
- Sustain pedal use [*F*(3, 16.59) = 3.297, *p* = 0.046, η^2^ = 0.133]: average time (in ms) during which the sustain pedal is depressed while a chord is being played.

The first eight performance features presented describe dynamics and attack. They reveal that Pianist A used the lowest dynamics (hammer velocity), attack speed and percussiveness, while featuring the longest attacks, and rather shallow key depressions. She also showed the largest variations in intensity between chords. On the other hand, Pianist C applied the highest intensity, fastest attacks and deepest key depressions. His attacks were the longest, but less saliently than his attack speeds could have led to believe. This may be explained by the rather average percussiveness of his attacks: with a pressed touch, key depression starts with zero velocity, which at equal hammer velocity and attack speed may inflate attack duration compared with a struck touch. Pianist C also presented the highest variations in attack duration, yet was the most constant in hammer velocity, which indicates he may have varied his touch percussiveness while always reaching high intensities. Pianist B's attacks are short, deep and the most percussive, as well as the most consistent in duration, yet they are not very fast, and produce low to average intensity (with average consistency). As for Pianist D, he played with average-to-high intensity, attack speed and percussiveness, with equally average-to-high variations in intensity between chords. His attacks were consistently short. Yet his key depressions were the shallowest. Furthermore, all four pianists applied higher intensities with the right hand, but to varying degrees (the least by Pianist C, the most by Pianist D). As a side note, these eight features of attacks and dynamics were significant (in the sense previously indicated) over the chords played by each hand separately, except for attack percussiveness and the difference in hammer velocity between hands (the latter ineligible). Of the six features, all but hammer velocity were more salient in left-hand chords than in right-hand chords, meaning that the differences in attack between the four pianists were more manifest in their left-hand playing than in their right-hand playing.

The following four performance features represented describe articulation, polyphonic touch, and tempo. The melody lead effect was longer on average for Pianist A than for the three others. Pianist A shows the most *legato* articulation and the longest key releases. On the other hand, the duration of her sustaining key depressions is about average, and inter-onset intervals are short, i.e., a fast tempo (actually close to score indications). Likewise, Pianist C played at a fast average tempo (as indicated on the scores) given his short inter-onset intervals. Yet his articulation is *non-legato*, with the shortest key releases and average durations of key sustains. Pianist B played extremely *staccato* compared with the others. His key releases remained of average duration, but his key sustains were the shortest, which left enough space for large intervals between chords despite the slowest tempo he favored. His preference for *staccato* articulation matches well with his short and percussive, yet not very fast, attacks. For Pianist D, key sustains and releases were very long, which may be mostly due to his long inter-onset intervals (slow tempo), whereas his articulation remains essentially *non-legato*.

Finally, the last three performance features describe pedaling. Pianist D used the soft pedal extensively (including part-pedaling), while Pianist C never used the soft pedal. Pianists A and B used the soft pedal sparingly, with some part-pedaling from A but none from B. The sustain pedal was also used extensively by Pianist D, as well as by Pianist A. Pianists B and C used the sustain pedal the least, yet still quite a bit.

#### 3.1.4. Timbre-pianist interaction

In complement to this characterization of the four pianists' general individuality over all performances (regardless of the timbral nuance expressed), we also aimed at determining the idiosyncrasies in pianists' performances that could arise specifically in the expression of each of the five timbral nuances considered. Separate statistical analyses (ANOVA) of performance features were performed to this aim, over each set of performances highlighting the same timbral nuance. However, these separate analyses are only valid for the performance features protected by a significant effect of the timbre-pianist interaction in the general ANOVA.

The effect of the pianist-timbre interaction, with regard to the 616 performance features, was thus examined in the general, three-Way repeated-measures ANOVA (with performer as random factor) over all performances. Violations of sphericity were corrected with the Huynh–Feldt epsilon applied to the degrees of freedom. The effect of the pianist-timbre interaction was significant (at the 5% level for corrected *p*-values) for 149 performance features.

Among these 149 performance features, 86 also showed a significant effect of the pianist. Yet the features of melody lead and variations in attack duration, included in the overall performance portrait as two of the most relevant descriptors of pianists' general individuality, were not found significant in the pianist-timbre interaction. For the variations in attack duration, the non-significant effect of pianist-timbre interaction is supported by non-negligible statistical power (π = 0.456), which may allow to infer that the variations in attack duration characterize general idiosyncratic traits that are not influenced by the timbral nuance performed. However, given the low statistical power of the non-significant pianist-timbre interaction for melody lead (π = 0.188), the same inference should not be made.

As for the features significant for the interaction but not for the effect of pianist, they will be discussed in relation with the timbral nuance(s) for which they bear a particular idiosyncratic effect.

### 3.2. Pianists' individuality in performing each of five timbral nuances

The performance strategies and performance features highlighting pianists' individuality in the production of five different timbral nuances were sought out, among the 149 performance features that showed a significant effect of pianist-timbre interaction in the general ANOVA over all performances.

Statistical analyses of variance were conducted separately over each set of 48 performances highlighting one of the five timbral nuances. Five Two-Way repeated measures ANOVAs were performed, with each of the 149 performance features as dependent variable, with the performer as random factor, and piece and repetition (of the same performer-×-piece condition) as fixed-effect factors.

Like in the overall case, for each ANOVA, the between-subject effect of performer was considered. The corresponding assumptions of normal distributions (Kolgomorov–Smirnov test) and homoscedasticity (Levene's test) were tested, and a non-parametric, one-way Kruskal–Wallis rank analysis of variance was performed to confirm or reject the significance of an ANOVA whose assumptions were not met. Effect sizes and statistical power were also calculated. Moreover, the within-subject effect of repetition was taken into account, with the degrees of freedom of the ANOVA corrected for violation of sphericity with the Huynh–Feldt epsilon. A performance feature was then considered as revealing pianists' individuality when the effect of performer was significant at the 5% level (in rejecting the null hypothesis of equal variance between pianists) while the effect of repetition was not (i.e., no significant differences between repetitions).

Consequently, for the sets of performances highlighting each of the bright, dark, dry, round, and velvety timbral nuances, significant differences between pianists and consistence between repetitions were identified in 86, 83, 69, 72, and 97 performance features (respectively).

#### 3.2.1. Reduced performance spaces

For each set of 48 performances highlighting one of the five timbral nuances, Principal Component Analysis was applied to the corresponding performance features selected, whose values were normalized (Z-score per feature over 48 performances).

In each of the five cases, the first two principal components were sufficient to explain a large part of the input variance:

- Bright timbre: 60.6% (41.7 and 18.9% respectively)
- Dark timbre: 60.6% (34.4 and 26.2% respectively)
- Dry timbre: 64.1% (45.1 and 19% respectively)
- Round timbre: 55.5% (39.5 and 16% respectively)
- Velvety timbre: 57.5% (39.9 and 17.6% respectively)

For each of the five timbral nuances, Varimax factor rotation of the first two PCA loadings was attempted, yet did not noticeably improve the clarity of the reduced performance spaces, nor provided a more optimal/interpretable distribution of loadings between features along each principal component, and was thus discarded.

**Figure 4:**
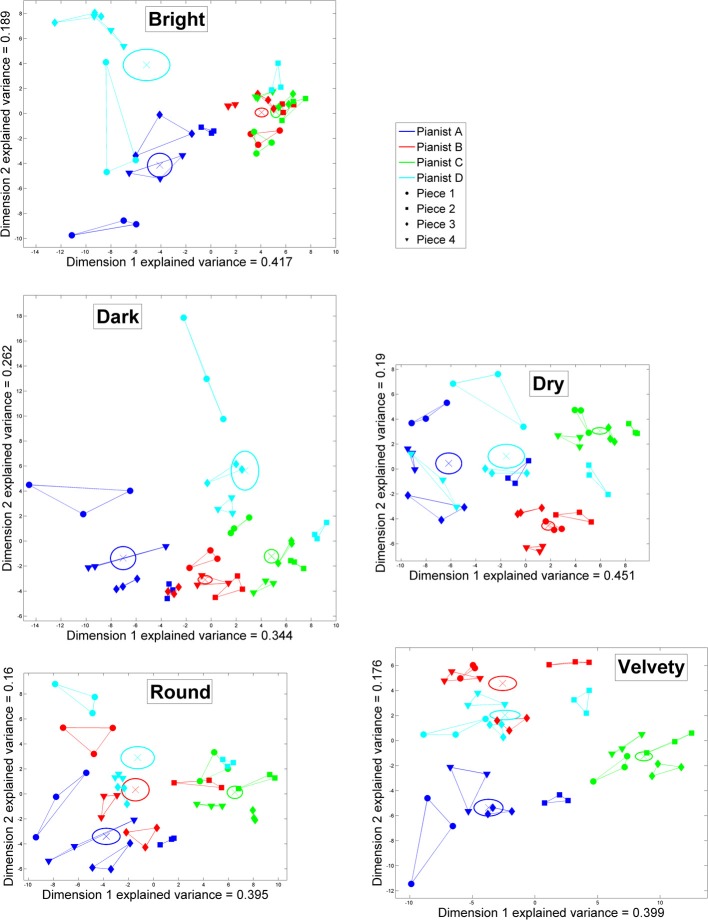
**Principal Component Analysis of the performance features highlighting pianists' individuality over each set of 48 performances corresponding to a different timbral nuance (bright, dark, dry, round, or velvety): two-dimensional reduced performance spaces**. In each of the five subplots, the colored lines link the same-piece repeated performances by each pianist. Averages per pianist are indicated by colored crosses, and ±1 SE with ellipses.

For each of the five timbral nuances, the position of each same-timbre performance according to its coordinates in the two dimensions formed by the corresponding first two principal components is presented in a separate plot in Figure 4.

For all five timbres, the loadings of the first dimensions primarily account for overall dynamics (hammer velocity) and attack speeds. For all timbres but dark, these features are also predominant with regard to the left hand, whereas for the dark timbre these features are more weighted for the right hand than for the left. Attack durations are also highly weighted, primarily with regard to the left hand (especially for the dark timbre). Variations in hammer velocity also largely contribute to the first dimension, overall and with regard to the left hand (but not the right), and especially for the bright timbre. Other features of touch and articulation contribute, to a lesser degree, to the first dimensions: key depression depth (bright, dry, round), attack percussiveness (dark, velvety), right-hand overlaps (bright); key release durations (round, velvety).

The descriptions of second dimensions according to loadings feature, for all timbres but bright, common trends of articulation, tempo, and pedaling, although in different combinations and weighting order. From highest to lowest weighted features, for dark: note and key sustain durations, sustain pedal use, soft pedal use, and articulation. For dry: sustain pedal use and articulation. For round: inter-onset intervals, sustain pedal use, note and key sustain durations, articulation. For velvety: articulation and soft pedal use. On the other hand, the second dimension for the bright timbre essentially corresponds to right-hand attack speeds and durations, then right-hand hammer velocities, and much less so to articulation.

It must also be mentioned that, in the reduced space of bright-timbre performances, soft pedal use is also accounted for in both dimensions (especially the second). However, only Pianist D used the soft pedal in bright-timbre performances, in the seven performances isolated in the upper left of the performance space. For the other 41 bright-timbre performances, soft pedal features bear no influence.

On average, Pianists B and C tend to be the most consistent for each timbre, both in same-piece repetitions and between pieces (with the exception of Pianist B's round-timbre performances). Difference between pianists are more or less salient depending on timbres and pianists. Pianists A and C are clearly differentiated for all timbres, as their performances occupy different regions of the performance spaces. Pianists A and D differ the most for timbres dark and velvety. Pianist B's dry-timbre performances are clearly singled out, yet for other timbres his performances overlap with different pianists. In particular, Pianists B and C's bright-timbre performances are all but indistinguishable in the corresponding performance, which means that they adopted equivalent strategies as regards the performance features relevant to the individuality-highlighting performance space of the bright timbre.

Moreover, some idiosyncratic tendencies revealed in the overall reduced performance space also appear in the timbre-wise reduced performance spaces, yet may be nuanced depending on the timbral nuance. Indeed, although Pianist A generally shows low dynamics, and slow and long attacks (along the first dimensions), this fact is more salient for dark-timbre performances (and dry to a lesser degree), while intensity, attack speeds and durations are more similar between Pianists A, B and D for round and velvety timbres. Pianists A and D are also quite similar in intensity, attack speeds and durations for a bright timbre, yet only along the first dimension. Indeed, the second dimension accounts for right-hand dynamics/attacks (and with the previously stated effect of soft pedal use by Pianist D in the seven upper-left performances notwithstanding) and highlights a difference between Pianist A and Pianist D's performances of piece no.2. On the other hand, Pianist C's high dynamics, and fast and short attacks, are most salient in velvety-timbre performances, and equivalent to Pianist B's for a bright timbre. Meanwhile, as the performance features associated with the second dimensions differ more largely between timbres, only timbre-wise tendencies can be brought up. For a bright timbre, differences between pianists can be explained by Pianist D's use of the soft pedal on one hand, and by Pianist A's lower right-hand dynamics/attacks on the other hand. For a dark timbre, Pianist D must have used longer notes and more sustain pedal, especially for piece no.1. For a dry timbre, Pianist B is probably set apart by his *staccato* articulation. For a round timbre, a conjecture is more complex to establish. Pianists A and D may be distinguished by tempo (fast vs. slow), while the same difference in tempo between Pianists B and C may be compensated by their different articulations. Finally, for a velvety timbre, articulation may suffice to explain the separation between Pianists A and B, while heavy soft pedal use may largely contribute to the higher-end coordinates of Pianist D's performances along dimension 2.

#### 3.2.2. Descriptive performance portraits

Following the same procedure as in the general case, the performance features most salient, relevant, meaningful, and non-redundant for highlighting pianists' individuality were sought out, for each of the bright, dark, dry, round, and velvety timbral nuances, among the 86, 83, 69, 72, and 97 (resp.) performance features selected for their eliciting significant differences between pianists and consistence between repetitions.

For each timbral nuance, the corresponding selected performance features were divided into the four functionally independent categories of dynamics/attack, articulation, soft pedal and sustain pedal. Correlations were calculated between all performance features within each category, and the most correlated/redundant features were grouped by hierarchical clustering. The most significant, meaningful and interpretable performance feature in each cluster was then selected.

**Figure 5:**
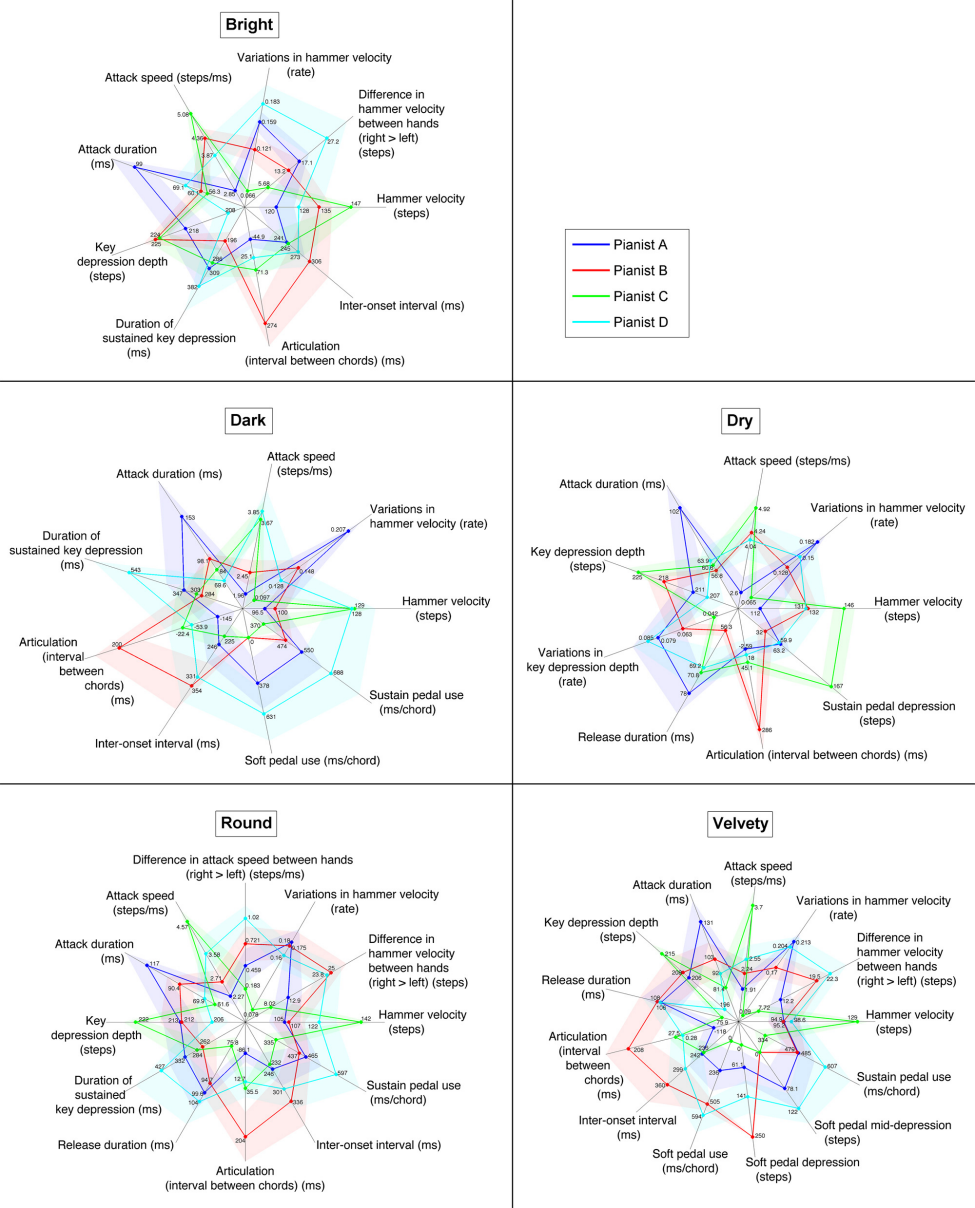
**Kiviats charts of the performance features giving a minimal and unique description of four pianists' individual performance patterns in the production of each of five timbral nuances (bright, dark, dry, round, and velvety)**. Z-scores per pianist are plotted for each feature with colored dots, with the corresponding unnormalized values indicated alongside. The four colored, dot-linking closed lines portray each pianist's performing style. Shades around each closed line show the ±1.96 SE. intervals (95% confidence interval).

For each of the bright, dark, dry, round, and velvety timbres, the 9, 9, 9, 12, and 13 (resp.) most relevant performance features for describing pianists' individuality were thus selected. The descriptive performance portraits of pianists' individuality for each timbral nuance are presented in Figure 5.

Most of the performance features used in these performance portraits of pianists' individuality in the production of each timbral nuance were already featured in the general performance portrait of pianists' individuality, and were accordingly described in Section 3.1.3. However, the following performance features were not previously introduced. In the performance portrait of individuality for a dry timbre:

- Variations in key depression depth: ratio of deviation between chords from the performance average of key depression depth.
- Sustain pedal depression: average depth (in 8-bit steps) of pedal depression per chord.

In the performance portrait of individuality for a round timbre:

- Difference in attack speed between hands: comparison of mean attack speed between notes played with the right hand and notes played with the left hand; values in steps/ms can indicate either faster (> 0) or slower (< 0) attacks with the right hand than the left.

In the performance portrait of individuality for a velvety timbre:

- Soft pedal depression: average depth (in 8-bit steps) of pedal depression per chord.

For each of the five performance portraits of the different timbral nuances, the statistical scores (ANOVA F-ratio, *p*-value and effect size) corresponding to each of its descriptive feature are provided below.

In the descriptive performance portrait of individuality for a bright timbre (9 features):

- Hammer velocity: *F*(3, 10.16) = 16.882, *p* = 2.8 · 10^−4^, η^2^ = 0.475
- Difference in hammer velocity between hands: *F*(3, 8.43) = 5.962, *p* = 0.018, η^2^ = 0.484
- Variations in hammer velocity: *F*(3, 10.84) = 16.060, *p* = 2.6 · 10^−4^, η^2^ = 0.703
- Attack speed: *F*(3, 10.85) = 24.475, *p* < 10^−4^, η^2^ = 0.660
- Attack duration: *F*(3, 10.66) = 8.415, *p* = 0.004, η^2^ = 0.581
- Key depression depth: *F*(3, 8.94) = 11.366, *p* = 0.002, η^2^ = 0.597
- Duration of sustained key depression: *F*(3, 9.97) = 5.504, *p* = 0.017, η^2^ = 0.221
- Articulation (interval between same-hand chords): *F*(3, 7.31) = 12.243, *p* = 0.003, η^2^ = 0.638
- Inter-onset interval: *F*(3, 8.03) = 5.463, *p* = 0.024, η^2^ = 0.089

In the descriptive performance portrait of individuality for a dark timbre (9 features):

- Hammer velocity: *F*(3, 11.74) = 22.588, *p* < 10^−4^, η^2^ = 0.638
- Variations in hammer velocity: *F*(3, 9.55) = 22.551, *p* = p = 1.2 · 10^−4^, η^2^ = 0.736
- Attack speed: *F*(3, 6.39) = 18.964, *p* = 0.001, η^2^ = 0.600
- Attack duration: *F*(3, 7.02) = 5.454, *p* = 0.030, η^2^ = 0.375
- Duration of sustained key depression: *F*(3, 9.33) = 10.297, *p* = 0.003, η^2^ = 0.343
- Articulation (interval between same-hand chords): *F*(3, 9.02) = 8.324, *p* = 0.006, η^2^ = 0.541
- Inter-onset interval: *F*(3, 12.21) = 7.169, *p* = 0.005, η^2^ = 0.280
- Soft pedal use: *F*(3, 8.54) = 5.876, *p* = 0.018, η^2^ = 0.516
- Sustain pedal use: *F*(3, 9.06) = 7.443, *p* = 0.008, η^2^ = 0.375

In the descriptive performance portrait of individuality for a dry timbre (9 features):

- Hammer velocity: *F*(3, 8.85) = 27.179, *p* < 10^−4^, η^2^ = 0.558
- Variations in hammer velocity: *F*(3, 3.57) = 28.319, *p* = 0.006, η^2^ = 0.634
- Attack speed: *F*(3, 9.40) = 24.816, *p* < 10^−4^, η^2^ = 0.619
- Attack duration: *F*(3, 10.43) = 7.148, *p* = 0.007, η^2^ = 0.535
- Key depression depth: *F*(3, 12.08) = 13.489, *p* = 3.7 · 10^−4^, η^2^ = 0.504
- Variations in key depression depth: *F*(3, 6.26) = 9.163, *p* = 0.011, η^2^ = 0.463
- Release duration: *F*(3, 10.14) = 7.132, *p* = 0.007, η^2^ = 0.433
- Articulation (interval between same-hand chords): *F*(3, 7.62) = 11.342, *p* = 0.003, η^2^ = 0.685
- Sustain pedal depression: *F*(3, 7.18) = 14.834, *p* = 0.002, η^2^ = 0.676

In the descriptive performance portrait of individuality for a round timbre (12 features):

- Hammer velocity: *F*(3, 9.02) = 35.453, *p* < 10^−4^, η^2^ = 0.589
- Difference in hammer velocity between hands: *F*(3, 8.12) = 9.802, *p* = 0.004, η^2^ = 0.548
- Variations in hammer velocity: *F*(3, 5.52) = 18.583, *p* = 0.003, η^2^ = 0.624
- Difference in attack speed between hands: *F*(3, 8.64) = 4.131, *p* = 0.044, η^2^ = 0.431
- Attack speed: *F*(3, 8.92) = 53.632, *p* < 10^−4^, η^2^ = 0.686
- Attack duration: *F*(3, 9.47) = 12.217, *p* = 0.001, η^2^ = 0.575
- Key depression depth: *F*(3, 8.98) = 12.875, *p* = 0.001, η^2^ = 0.470
- Duration of sustained key depression: *F*(3, 9.22) = 7.350, *p* = 0.008, η^2^ = 0.211
- Release duration: *F*(3, 10.60) = 6.136, *p* = 0.011, η^2^ = 0.376
- Articulation (interval between same-hand chords): *F*(3, 9.38) = 9.385, *p* = 0.004, η^2^ = 0.569
- Inter-onset interval: *F*(3, 10.91) = 7.121, *p* = 0.006, η^2^ = 0.188
- Sustain pedal use: *F*(3, 10.40) = 8.627, *p* = 0.004, η^2^ = 0.387

Finally, in the descriptive performance portrait of individuality for a velvety timbre (13 features):

- Hammer velocity: *F*(3, 5.17) = 92.653, *p* < 10^−4^, η^2^ = 0.606
- Difference in hammer velocity between hands: *F*(3, 9.34) = 11.909, *p* = 0.002, η^2^ = 0.650
- Variations in hammer velocity: *F*(3, 6.64) = 35.600, *p* = 1.8 · 10^−4^, η^2^ = 0.789
- Attack speed: *F*(3, 4.13) = 69.362, *p* = 5.5 · 10^−4^, η^2^ = 0.635
- Attack duration: *F*(3, 8.76) = 10.662, *p* = 0.003, η^2^ = 0.436
- Key depression depth: *F*(3, 8.11) = 25.073, *p* = 1.9 · 10^−4^, η^2^ = 0.575
- Release duration: *F*(3, 5.87) = 14.320, *p* = 0.004, η^2^ = 0.489
- Articulation (interval between same-hand chords): *F*(3, 7.41) = 12.912, *p* = 0.003, η^2^ = 0.624
- Inter-onset interval: *F*(3, 8.84) = 9.085, *p* = 0.005, η^2^ = 0.245
- Soft pedal use: *F*(3, 8.93) = 11.448, *p* = 0.002, η^2^ = 0.613
- Soft pedal depression: *F*(3, 10.98) = 20.321, *p* < 10^−4^, η^2^ = 0.759
- Soft pedal mid-depression: *F*(3, 10.00) = 6.684, *p* = 0.009, η^2^ = 0.542
- Sustain pedal use: *F*(3, 7.53) = 8.579, *p* = 0.008, η^2^ = 0.365

The complete list of performance features selected as significant, consistent, meaningful and non-redundant in highlighting pianists' individuality in the production of at least one timbral nuance and/or overall is presented in Table 1. For each performance feature and each timbral nuance, the table indicates whether the feature was selected in the descriptive portrait, or significant but redundant with others, or not protected by a significant effect of timbre-pianist interaction in the general ANOVA, or whether non-significance was conclusive (with regard to statistical power).

**Table 1:** **Performance features most characteristic of pianists' individuality, in performing five timbral nuances and overall**.

**Timbre: Performance features**	**Bright**	**Dark**	**Dry**	**Round**	**Velvety**	**All**
**ATTACK AND DYNAMICS**						
Hammer velocity	O	O	O	O	O	O
Difference in hammer velocity between hands	O	×	–	O	O	O
Variations in hammer velocity	O	O	O	O	O	O
Attack speed	O	O	O	O	O	O
Difference in attack speed between hands	S	–	×	O	×	×
Attack percussiveness	×	S	–	×	S	O
Attack duration	O	O	O	O	O	O
Variations in attack duration	.	.	.	.	.	O
Key depression depth	O	–	O	O	O	O
Variations in key depression depth	S	**–**	O	×	×	S
**ARTICULATION**						
Articulation (intervals between chords)	O	O	O	O	O	O
Duration of sustained key depression	O	O	×	O	×	O
Release duration	×	×	O	O	O	O
Inter-onset interval	O	O	×	O	O	O
Melody lead	.	.	.	.	.	O
**PEDALS**						
Soft pedal use	S	O	S	X	O	O
Soft pedal depression	S	×	×	X	O	–
Soft pedal mid-depression	S	S	×	S	O	O
Sustain pedal use	×	O	X	O	O	O
Sustain pedal depression	×	–	O	–	–	–

*The following symbols were used for: O, feature included in the descriptive portrait of the timbral nuance; S, feature significant in highlighting pianists' individuality, but redundant with others; “.” (dots), feature not protected by the pianist-timbre interaction in the general ANOVA; the other features were not significant with regard to individuality, “−” (dashes), feature non-significant with regard to individuality, with low statistical power (π < 0.2), thus inconclusive; x; id., with average statistical power (0.2 < π < 0.8); X: id., with high statistical power (π > 0.8), thus conclusive*.

Some of the pianists show consistent patterns across timbres along some of the performance features, which mostly reflect the general descriptive portrait, and also correspond to what could be deduced from the reduced performance spaces. Pianist A always presents the lowest hammer velocities, and the longest and slowest attacks. On the other hand, Pianist C always produced the highest and most regular hammer velocities, as well as fast and short attacks. In the four timbral nuances (all but dark) for which key depression depth was selected as a performance significant for pianists' individuality, Pianist C always applied very deep key depressions (close to the keybed), while on the other hand Pianist D always employed the shallowest key depressions. On average, for each of the four timbral nuances, maximum key depressions per note were approximately 10% deeper for Pianist C than for Pianist D. As for articulation, Pianist A always played with the most *legato*, and Pianist B with the most *staccato*. Key depression sustains were also consistently the longest for Pianist D, and the shortest for Pianist B, although this performance feature was only significant in highlighting individuality for three of the five timbral nuances (bright, dark, and round). Finally, inter-onset intervals could significantly portray the pianists' individuality in performing four timbral nuances (all but dry), and were larger for Pianist B (and Pianist D to a lesser degree) than for Pianists A and C, indicating the same differences in average tempo as previously described in the general case over all performances.

Yet otherwise, different performance patterns between pianists arose along performance features in the production of different timbral nuances. Dynamic balance between hands (in hammer velocity) was only significant in the production of bright, round and velvety timbres (although its non-significance for a dry timbre was inconclusive due to low statistical power). Pianist D always largely emphasized the right hand, while Pianist C always used the least right-hand emphasis. Pianist B drastically changed his right-hand dynamic emphasis between timbres, from average (among the four pianists) for bright and velvety timbres, to the most of all for round. Balance between hands in attack speed was also selected as a relevant feature for describing individuality in the production of a round timbre. In contrast with the corresponding dynamic balance between hands, the discrepancy in attack speed between hands (toward the right) for a round timbre is larger (and largest) for Pianist D than Pianist B, although the latter shows a more pronounced dynamic emphasis on the right hand. Dynamic variations within a performance also largely changed between timbres for Pianists A, B, and D. Pianist A's dark performances always featured high dynamic variations, and by far the most of all pianists. On the other hand, Pianist D used even more dynamic variations than her for the production of a bright timbre. Pianist D's dynamic variations are also high for dry, round, and velvety timbres, yet below average for dark. Meanwhile, Pianist B's dynamic variations range from average among pianists for bright, dark and dry timbres, to very high for round performances. Patterns of hammer velocities, attack speeds and durations also change between timbres for Pianists B and D. For a bright timbre, Pianist D's attacks are about average in intensity, speed and duration, while Pianist B's attacks are faster, especially shorter, and brought higher hammer velocities, both than average and than Pianist D's. For the dry timbre, Pianists B and D's attacks are mostly equivalent in intensity, speed and duration, yet although their hammer velocities and attack speeds are average and well below Pianist C's, their attacks are among the shortest (with Pianist C's). For Pianist B, these shortened attacks (despite average speeds) can be explained by his preferred *staccato* articulation, while for Pianist D the same attack characteristics may stem from his shallow key depressions. On the other hand, for round and velvety timbres, Pianist B's attacks are slower and longer than average, while Pianist D's attacks are faster and shorter than Pianists B's and than average (especially for round). These patterns result for Pianist D in hammer velocities average for round, yet quite lower for velvety, while for both timbres Pianist B's hammer velocities are the lowest along with Pianist A's. Finally, for producing a dark timbre, Pianist B remains around low intensities and slow/long attacks, whereas Pianist D employed the fastest and shortest attacks, and the (nearly) highest hammer velocities of all four pianists.

As for key depression depths, besides the consistently deep vs. shallow key depressions for Pianists C and D (resp.), both applied slightly shallower key depressions (while still differing from each other by about 10%.) in performing a velvety timbre than for the other three nuances (bright, dry and round) for which key depression depth was a relevant feature of individuality (key depression depth was inconclusively non-significant in highlighting pianists individuality in dark-timbre performances). While Pianist A remained fairly constant, and average among pianists, in average key depression depth per timbral nuance, Pianist B varied the most in key depression depth between timbral nuances, both in the absolute and with regard to the other pianists, ranging from the deepest key depressions (in performing a bright timbre) to average ones (in performing a round timbre). Moreover, variations in key depression depth (within a performance) were only relevant feature for individuality in the case of a dry timbre. The pianists with the shallower average key depressions (A and D) also show the largest variations (and vice versa), which might be due (at least in part), to a ceiling effect (key depressions cannot get deeper than the keybed; the deeper the key depressions on average, the less room there remains for variations).

Articulation patterns also reflect a different picture of pianists' individuality depending on the timbral nuance expressed.

Key depression sustains were indeed much shorter for Pianist B (than the three others) in the case of a bright timbre, whereas their durations were much closer between Pianists B and C (and Pianist A to a lesser degree) for timbres dark and round.

Key release duration, a significant descriptor of individuality in the production of the three timbres dry, round and velvety, highlighted very different patterns between pianists depending on the timbral nuance. Although Pianist A always featured long key releases, they were only the longest (or more appropriately, the least short) with a dry timbre. Pianist B's key releases were the shortest for a dry timbre, yet the longest for velvety. Pianist D's key releases were also very long (relative to the other pianists) for velvety and especially round timbres, yet only average (and much shorter in the absolute) for dry. On the other hand, Pianist C's key releases, the shortest for round and velvety timbres, were only average among pianists for the dry timbre, although they remained of fairly constant duration in the absolute between timbres (i.e., contrary to the others pianists, Pianist C did not use or show different key release durations in his performing the three different timbral nuances dry, round and velvety).

Articulation, as described by the timing intervals between same-hand chords, presents mostly consistent patterns of individuality between four of five timbral nuances, yet for a dry timbre Pianist A's articulation is not significantly more *legato* than Pianists C and (especially) D, as all three feature an articulation best described as *non-legato* in performing a dry timbre.

Furthermore, the difference in inter-onset intervals (i.e., average tempo) between Pianists B/D and A/C was much more salient for the dark timbre than for round, velvety and (especially) bright.

Lastly, the differences in pedaling strategies between pianists largely vary depending on the timbral nuance performed. In performing a velvety timbre, the soft pedal was used by Pianists A (sparingly), D (massively), and B (constantly, in all velvety performances). Meanwhile, only Pianists A and D used the soft pedal in performing a dark timbre (both to the same extent as for velvety). For the other three timbral nuances, the soft pedal (although it is not presented in the descriptive portraits) was only used in some of Pianist D's performances, and never by the three other pianists.

Finally, the duration of sustain pedal use with regard to chords was significant for timbres dark, round, and velvety, and although idiosyncratic patterns were largely consistent between timbres, with the most use for Pianist D, the least for Pianist C, and average relative use for Pianist A, Pianist B tended to use more sustain pedal (especially with regard to Pianist A) from timbres dark to velvety. Although sustain pedal use was conclusively non-significant in highlighting pianists' individuality for a dry timbre, the amount of sustain pedal depression was significant, and especially highlighted an higher amount of depression for Pianist C.

## 4. Discussion

In summary, individual strategies between four pianists were successfully revealed, within 240 performances of four pieces with five different timbral nuances, by the fine-grained performance control features extracted from the high-accuracy key, pedal and hammer-tracking data gathered by the CEUS system. The four pianists were thus shown to elicit idiosyncratic patterns of dynamics, attack speed, attack touch (percussiveness, duration, depth, melody lead), articulation, key sustains and releases, average tempo, and pedaling.

Among these performance parameters, some would have been accessible with more rudimentary MIDI (or equivalent) data acquisition. Yet further subtleties of touch, articulation, and pedaling could only be revealed through continuous, high-accuracy key/pedal position tracking data. Although it cannot be determined from this study whether such performance subtleties bear a direct influence on sound production at the piano, they remain inherent to the process of piano playing, and (at least) indirectly involved (through mechanical, physiological, or kinaesthetic functions) in the actual sound production. These subtle control features may thus be considered as valid and valuable descriptors of individuality in piano performance.

In a broad characterization, the individual playing styles of the four pianists show the following salient traits:

- Pianist A's performances had characteristically the lowest dynamics (and high dynamic variability), the longest attacks, the least percussive touch, the longest melody leads (voice accentuation), and the most *legato* articulation—perhaps typical of a French playing style.
- Pianist B favored a very *staccato* articulation, short key sustains despite his playing at the slowest average tempo, and the most percussive touch. This playing style may be related to his upbringing in an Italian piano school that promotes detached playing.
- Pianist C's playing was essentially characterized by the highest (and most steady) intensity, the fastest attacks (yet not as percussive and barely the shortest), and the deepest key depressions. His articulation remained essentially *non-legato*, but with short key releases. He also made a personal choice in never using the soft pedal.
- Lastly, Pianist D's playing was mostly idiosyncratic in his heavy use of both pedals. His playing was also marked by a heavy dynamic emphasis of the right hand, and by generally shallow key depressions. His articulation remained *non-legato*, despite the longest key depression sustains.

Furthermore, with regard to the main hypothesis explored in this study, it was found that pianists' individuality expressed itself differently depending of the timbral nuance performed—within the general frame of their overall performance individuality. In other words, in addition to the general differences between the four pianists and the differences between each timbral nuance (common to all four pianists), the pianists also used some different performance strategies in order to highlight each timbral nuance.

Indeed, amid significant individual differences overall, dynamics were also changed differently by the four pianists in performing different timbral nuances (most saliently for dark-timbre performances, as only Pianist D did not lower his dynamics). Likewise, dynamic variations and balance between hands, as well as attack speed and depth, were altered differently by each pianist between timbral nuances. Articulation was also changed differently by each pianist between timbral nuances, especially in dry-timbre performances (in comparison with the four other timbres), where the tendency toward more *staccato* playing was followed to quite different degrees by each pianist. Finally, the use of the soft pedal was also specific to certain combinations of pianist and timbre.

On the other hand, some performance patterns that are essentially common to all four pianists in the production of different timbral nuances bear some similarity to those highlighted in the expression of different emotions. Given the resembling patterns of intensity and articulation between the strategies of timbre production and those of emotional expression, we may infer that the descriptors of piano timbre may possess some degree of correspondence with verbal descriptors of basic emotions. Velvety and dark timbres may thus be related to sad or tender emotions (low intensity, *legato* articulation), while a dry timbre may reflect happiness (high intensity, *staccato* articulation). This assumption may be supported by comments from two of the four participant pianists, who mentioned that, although undeniably valid and relevant as timbre descriptors, some of the five terms used (especially dry and dark) can actually double as descriptors of musical character—thus closer to reflecting an actual emotional imprint. However, this suggested correspondence between piano performances guided by descriptors of timbre and of emotion only relies on limited (MIDI-accessible) performance parameters. These are far from encompassing the more elaborated descriptions of the performances of different timbral nuances, which may pose the problem of a selection bias in picking the parameters to compare, and leaves several different ways in which the production of timbral nuances may stray from their emotional counterparts.

In conclusion, a novel approach was employed, in which a particular attention was given to respecting a valid musical context, with cohesive, original musical pieces composed for the study, and with timbral expression guided by verbal descriptors shown to the lexicon of piano timbre nuances of common, consensual and meaningful use among pianists. The first hypothesis that pianists' individuality could be revealed in subtle performance control features was confirmed. Moreover, the patterns of individual strategies and differences between the four pianists were found to differ, within the general frame of each pianist's individuality, between the production of different timbral nuances, thus confirming the second hypothesis.

### 4.1. Statistical validity of the findings

Some choices were made in the statistical analysis process. First, the significance levels of the ANOVAs were not adjusted for multiple comparisons, despite the 616 dependent variables tested overall and the 149 dependent variables tested per timbral nuance. Indeed, the corrections for multiple comparisons such as Bonferroni-Dunn or Holm-Bonferroni tend to be too conservative, especially when the assumption of independence between dependent variables is not met (as is the case in this study). Consequently, the risk of type II errors was reduced by not using a correction for multiple comparisons. However, we acknowledge the increased possibility of type I errors. Yet the analytic procedures used subsequently were designed so as to significantly reduce (if not eliminate) the risk of misinterpreting the results due to type I errors. Indeed, the contribution of falsely significant features to Principal Component Analysis can be considered as noise (i.e., more or less uniformly distributed between performers), and do not affect the characteristics of pianists' individuality in the corresponding performance spaces—only their legibility. Moreover, the descriptive performance portraits were obtained after careful selection of only one feature within each cluster of highly-correlated features. As the effect size, significance level, and corresponding statistical power in highlighting pianists' individuality were all considered for selecting only the best such feature per cluster, it is improbable that falsely significant features may appear in the descriptive portraits. Moreover, for some clusters where no variable could meet minimum thresholds for both effect size and statistical power (η^2^ < 0.2, π < 0.2), no feature was selected.

Furthermore, we opted to perform separate ANOVAs for each timbral nuance, instead of relying on pairwise *post-hoc* tests of the pianist-timbre interaction in the general ANOVAs, as the latter can be too conservative, and especially less informative, as they do not distinguish the two-dimensional structure of the interaction effect, i.e, treat all pairs of interaction cases equally (disregarding whether they correspond to the same pianist or timbre). The solution we privileged thus allowed for a more exhaustive exploration and account of the pianist-timbre interaction (i.e., the expression of pianists' individuality in the production of different timbral nuances.

### 4.2. Perspectives

The results presented in this article can be set in perspective with the common performance control strategies adopted by the four pianists in order to produce and express the five different timbral nuances, which were explored in Bernays and Traube (2013).

On the other hand, it cannot be determined from this study whether the observed differences in individual performance strategies between the five timbral nuances stem from a different understanding among the four pianists of the timbre descriptors used as performance instructions, or characterize different ways of reaching a common timbral idea. However, the perception and identification of timbre in the audio recordings of the performances analyzed in this article has been investigated (Bernays, 2013). Preliminary results suggest that the different timbral nuances expressed in the performances can be reliably identified (significantly above chance level) by other pianists, which would indicate that, for each of the five timbral nuances, the differences in performance strategy between the four performers may have yielded, despite possible audible differences in sound production, the same perceptual effect as regard timbre identification.

A parallel may be drawn with vocal expression, where non-verbal affective cues remain consistently identifiable between different speakers (even in single vowels), despite large acoustic inter-individual differences, especially in voice quality (i.e., timbre) (Juslin and Scherer, 2005). Likewise, despite inter-individual differences, the timbral nuances expressed at the piano may remain identifiable, and may be categorized according to corresponding adjectival descriptors.

Furthermore, the four musical pieces used in this study were expressly chosen for their different musical characteristics and the different playing styles they would require. Consequently, the performances of all four pieces were analyzed conjointly, with the statistical the effect of the musical piece performed separated from the effects of performer and timbre, in the aim of highlighting individuality in piano performance over an extended, representative set of musical characteristics. It may be worthwhile then to further disentangle the individual playing styles of pianists from the influence of pieces and interpretive goals, as was accomplished by Gingras et al. (2013) in the context of harpsichord performance, by likewise using the analysis methods of complete linear mixed models and correlation-based similarity profiles. The relations between pianists' individuality (as revealed by characteristic performance features) and the musical structure of each piece performed may also be investigated with the software tools included in the PianoTouch toolbox (Bernays and Traube, 2012), in order to assess pianists' individuality as a function of the different musical contexts featured in the pieces.

Finally, the effect of the performance features characteristic of pianists' individuality upon the actual sound production may be explored with acoustical analyses of the audio recordings of the performances.

In the end, this research may help determine the expressive boundaries of individual piano performance within which the same timbral nuance can be produced and perceived.

## 5. Funding

FRQSC (*Fonds de Recherche du Québec – Société et Culture*); CIRMMT (Centre for Interdisciplinary Research in Music Media and Technology); OICRM (*Observatoire Interdisciplinaire de Création et de Recherche en Musique*).

### Conflict of interest statement

The authors declare that the research was conducted in the absence of any commercial or financial relationships that could be construed as a potential conflict of interest.
